# Transgenic expression of cyclooxygenase-2 (COX2) causes premature aging phenotypes in mice

**DOI:** 10.18632/aging.101060

**Published:** 2016-10-07

**Authors:** Joohwee Kim, Vivek Vaish, Mingxiao Feng, Kevin Field, Ioulia Chatzistamou, Minsub Shim

**Affiliations:** ^1^ Department of Biological Sciences, University of South Carolina, Columbia, SC 29208, USA; ^2^ Center for Colon Cancer Research, University of South Carolina, Columbia, SC 29208, USA; ^3^ Department of Pathology, Microbiology & Immunology, School of Medicine, University of South Carolina, Columbia, SC 29209, USA

**Keywords:** premature aging, COX2, TP53, p16, prostaglandin

## Abstract

Cyclooxygenase (COX) is a key enzyme in the biosynthesis of prostanoids, lipid signaling molecules that regulate various physiological processes. COX2, one of the isoforms of COX, is highly inducible in response to a wide variety of cellular and environmental stresses. Increased COX2 expression is thought to play a role in the pathogenesis of many age-related diseases. COX2 expression is also reported to be increased in the tissues of aged humans and mice, which suggests the involvement of COX2 in the aging process. However, it is not clear whether the increased COX2 expression is causal to or a result of aging. We have now addressed this question by creating an inducible COX2 transgenic mouse model. Here we show that post-natal expression of COX2 led to a panel of aging-related phenotypes. The expression of p16, p53, and phospho-H2AX was increased in the tissues of COX2 transgenic mice. Additionally, adult mouse lung fibroblasts from COX2 transgenic mice exhibited increased expression of the senescence-associated β-galactosidase. Our study reveals that the increased COX2 expression has an impact on the aging process and suggests that modulation of COX2 and its downstream signaling may be an approach for intervention of age-related disorders.

## INTRODUCTION

Cyclooxygenase (COX) is a rate-limiting enzyme in prostanoid biosynthesis [1]. Prostanoids, including the prostaglandins, prostacyclin and thromboxane, are lipid signaling molecules that regulate numerous physio-logical processes such as inflammation, pain mediation, renal function, reproduction, and gastrointestinal integrity [2]. COX catalyzes the conversion of arachidonic acid to prostaglandin H_2_ (PGH_2_), which is then converted to various prostanoids by specific prostanoid synthases. Once synthesized, prostanoids are released outside the cells and bind to their specific G-protein coupled receptors (GPCRs) in an autocrine or paracrinemanner, stimulating various downstream signaling molecules.

There are two isoforms of COX: COX1 and COX2. These enzymes catalyze a nearly identical reaction and are inhibited by NSAIDs. However, their expression pattern is distinctly different. COX1 is constitutively expressed in many tissues and is thought to play a role in tissue homeostasis. In contrast, COX2 has a low or undetectable level of expression in most normal tissues and cell types. However, its expression is highly induced by bacterial products or pro-inflammatory cytokines, suggesting that COX2 plays a role in inflammatory responses. In addition, COX2 expression is highly induced by various environmental stressors such as UV light, cigarette smoke, diet, heavy metals, and xenobiotics [3–6]. Therefore, increased expression of COX2 may be an indicator of cellular and environmental stress. Consistent with this, COX2 expression is increased in many human diseases. Increased COX2 expression from cellular and environmental stress is often in turn translated into a pathological outcome. Numerous human and mouse studies have demonstrated the role of COX2 in the development and/or progression of various diseases [7].

A general characteristic of the aging process is a progressive decline of physiological functions, leading to an inability to maintain cellular homeostasis. As mentioned earlier, COX2 is involved in the development and/or progression of many age-related diseases including atherosclerosis, arthritis, cancer, diabetes, osteoporosis, and Alzheimer's disease [8–14]. Moreover, studies have reported that COX2 expression is increased during aging. For example, in aged rodents, COX2 expression is up-regulated in the heart, prostate, liver, kidney, and macrophages [15–19]. Increased COX2 expression was also reported in aging human tissues such as skin, mononuclear cells, and kidney [20–23]. However, it is not clear whether the increased COX2 expression is causal to or a result of aging.

We developed a COX2 transgenic mouse model and previously reported that embryonic expression of COX2 causes fetal malformations and post-natal death [24]. To examine how aberrant COX2 expression interferes with normal physiological processes in adult animals, we generated an inducible COX2 transgenic mouse model. In the present study, we report multiple phenotypic changes and pathologies, which are characteristic of premature aging, in COX2 transgenic mice. Our study suggests that up-regulation of COX2 may play a role in the decline of tissue function during aging and in the pathophysiology of age-related diseases.

## RESULTS

### Generation of inducible COX2 transgenic mice

**Figure 1:**
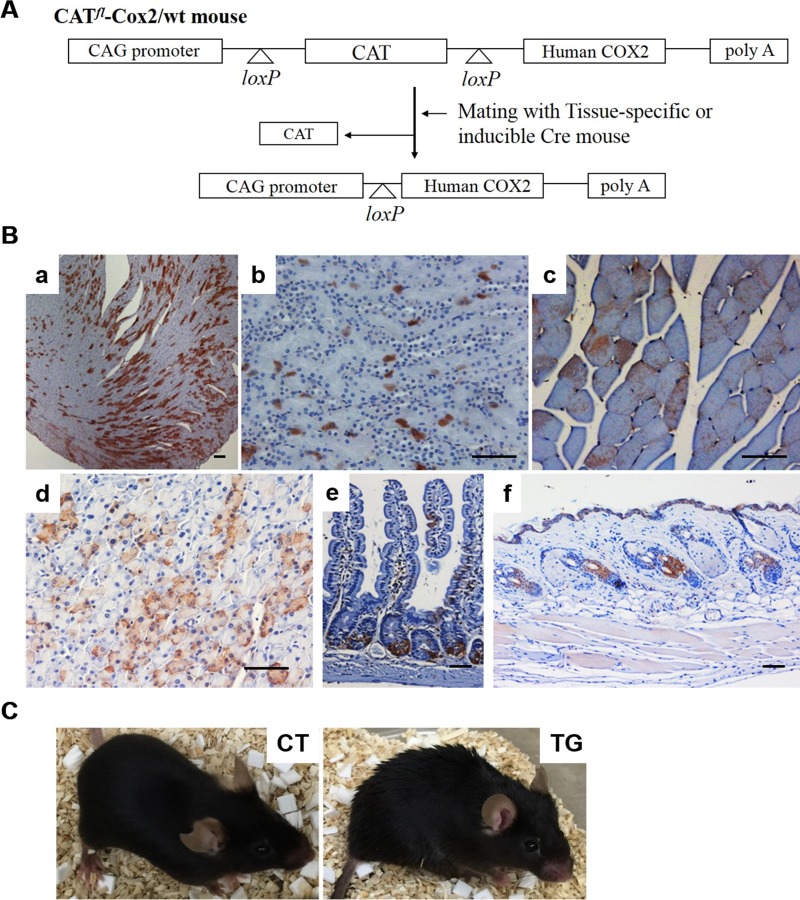
(**A**) Generation of tamoxifen-inducible COX2 transgenic mouse. Diagram of CAG/CAT/hCOX2 construct is shown. (**B**) Expression of COX2 in various tissues of tamoxifen-inducible COX2 transgenic mouse. COX2 expression was induced in 5-week-old (CAT^fl^COX2:Rosa-Cre ERT2) mice by intra-peritoneal injections of tamoxifen. Paraffin-embedded sections of 20-week-old COX2 transgenic mice were stained for COX2 using a human COX2-specific antibody. The tissues shown are heart (*a*), kidney (*b*), skeletal muscle (*c*), pancreas (*d*), small intestine (*e*), and skin (f). Scale bar=100 μm (**C**) General appearance of 20-week-old control (CT) and COX2 transgenic (TG) mice.

A diagram of the COX2 transgene is shown in Fig. 1A. In this construct, the cytomegalovirus early enhancer/chicken β-actin (CAG) promoter directs the expression of the human COX2 gene. However, the expression of human COX2 gene is blocked by a chloramphenicol acetyl transferase (CAT) reporter gene flanked by two loxP sites. Two independent lines of floxed CAT/COX2 mouse (herein designated as *CAT^fl^COX2*) were produced. Since embryonic expres-sion of COX2 causes various malformations and post-natal lethality [24], we generated an inducible COX2 transgenic mouse model. CAT^fl^COX2 mice were crossed with ROSA-Cre ERT2 mice carrying a Cre recombinase-estrogen receptor-T2 allele targeted to the ubiquitously expressed ROSA26 locus [25]. Tamoxifen-inducible, Cre-mediated recombination at loxP sites results in deletion of the interfering CAT gene and, thus, human COX2 is expressed under the control of the CAG promoter in the COX2 transgenic mouse, which has both floxed COX2 and Cre-ERT2 alleles. COX2 transgenic mice without tamoxifen treatment appeared to be normal and were born at the expected Mendelian ratio (data not shown). To explore the effect of COX2 expression in adult mice, 5~6-week-old COX2 transgenic and control littermates were intra-peritoneally injected with tamoxifen. As shown in Fig. 1B, mosaic expression of COX2 was observed in the tissues of 20-week-old COX2 transgenic mice, including the heart, skin, muscle, pancreas, small intestine, and kidney. The expression of human COX2 transgene was not detected in non-induced COX2 transgenic mice (data not shown).

### Aging phenotypes in COX2 transgenic mice

**Figure 2:**
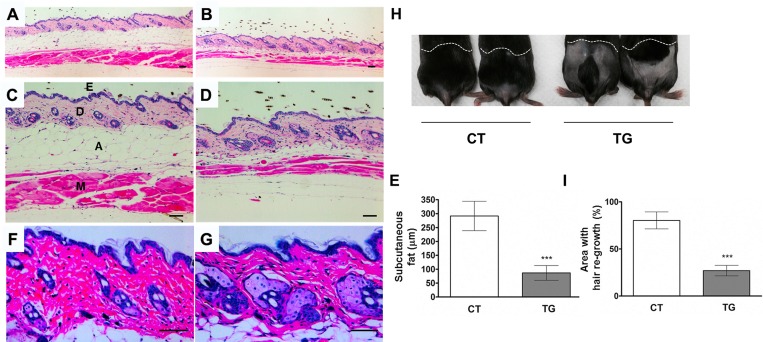
Cross-sections of dorsal skin from 20-week-old control (**A, C, a**nd **F**) and COX2 transgenic (**B, D,** and **G**) female littermates. Epidermis (E), dermis (D), adipose under the dermis (A), and muscle (M) are indicated. The COX2 transgenic skin exhibits sebaceous gland hyperplasia and a decrease in subcutaneous fat and muscle. Scale bar=100 μm (**E**) Quantification of the thickness of subcutaneous fat in control and COX2 transgenic mice (***: p<0.001). (**H**) A representative photo of 20- week-old COX2 transgenic and age-matched control male mice at 20 days after hair removal on dorsal area. (**I**) Quantification of hair re-growth in control and COX2 transgenic mice (***: p<0.001).

Tamoxifen-injected COX2 transgenic mice appeared normal until the age of 20 weeks, when we first noticed an unkempt and greasy hair coat (Fig.1C). Reduced subcutaneous fat is one of the markers for aged skin [26]. The analysis of H&E-stained sections of skin revealed a reduction in the subcutaneous fat in 20-week-old COX2 transgenic mice when compared to the skin in tamoxifen-injected control littermates (Fig. 2A-E, n=12). Additionally, hyperplasia of the sebaceous gland (Fig. 2F and G) was observed in COX2 transgenic skin, as is also observed in aged human skin [27, 28] and in the skin of XPD mutant mice, which exhibit premature aging phenotypes [29]. Hair re-growth also declines as a function of age in mice [30] and is reduced in other mouse models of aging [26, 31]. Thus, a hair re-growth assay was performed to investigate the impact of COX2 on hair growth. In mouse skin, coordinated hair regeneration occurs in a wave-like pattern across the skin surface. However, in old mice, waves of hair growth slow down, or hair cycle domains fragment into smaller domains, resulting in patchy hair growth [32]. As shown in Fig. 2H and I, delayed or patchy re-growth of hair was observed in all COX2 transgenic mice, whereas the age-matched control mice exhibited normal hair re-growth 20 days after removal of hairs.

**Figure 3:**
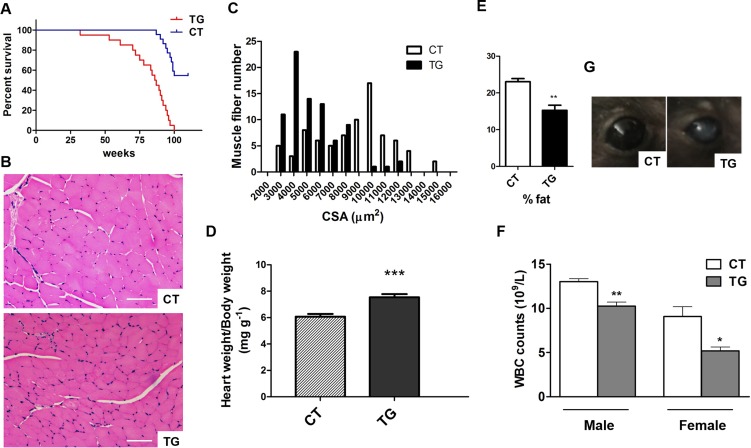
(**A**) Longevity in control (n=23) and COX2 transgenic (n=25) mice. (**B**) H&E stained sections of hind leg skeletal muscle from 22-week-old control (CT) and COX2 transgenic (TG) female littermates. Cross sections of the extensor digitorum longus (EDL) muscle are shown. Scale bar=100 μm (**C)** Frequency distribution of skeletal muscle fiber cross-sectional area (CSA) between control (CT) and COX2 transgenic (TG) mice. (**D**) Heart weight/body weight (HW/BW) ratios in tamoxifen-induced control (CT) and COX2 transgenic (TG) male littermates at the age of 22 weeks. HW/BW ratios in COX2 transgenic mice were significantly higher than those in control mice (paired t-test, p<0.001). (**E**) Quantitative assessment of fat content by X-ray densitometry of control (CT) and transgenic (TG) mice (n=5). (**F**) White blood cell (WBC) counts in tamoxifen-induced control (CT) and COX2 transgenic littermates (paired t-test, *: p<0.05, **: p<0.01, n=4). (**G**) The opacity of cornea developed in COX2 transgenic (TG) mice.

Tamoxifen-induced COX2 transgenic mice exhibited other signs of aging. The lifespan of COX2 transgenic mice was significantly reduced compared to that of control mice (Fig 3A). Studies have shown that there is a reduction in muscle fiber size with increasing age [33]. As shown in Fig. 3B and C, the size of the muscle fiber in the hind leg of COX2 transgenic mice was reduced compared to that in control littermates (n=5). Increase in heart weight is normally found in the aging human heart [34, 35]. The heart weight relative to body weight was increased in COX2 transgenic mice (Fig. 3D, n=9) compared to that in control littermates. We also performed quantitative assessments of body composition with X-ray densitometry of the whole mouse. The fat content was reduced in COX2 transgenic mice compared to control littermates, consistent with histological analysis of subcutaneous fat (Fig. 3E, n=5). However, bone mineral density was not significantly different between control and transgenic littermates (data not shown). COX2 transgenic mice also exhibited changes in peripheral blood composition. Total blood cell counts in 25-week-old transgenic and control mice show no differences in the number of red blood cells (data not shown). However, white blood cell counts in transgenic animals were significantly lower than those in control littermates (Fig. 3F). In addition, some COX2 transgenic mice developed ocular abnormalities such as opaque eyes and blindness (Fig. 3G).

**Figure 4:**
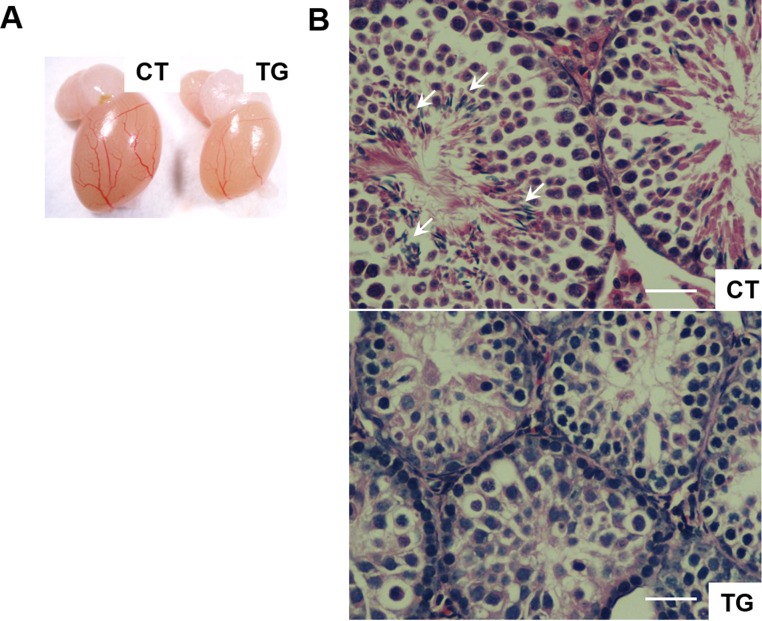
(**A**) A representative photo of testis from 23-week-old control (CT) and COX2 transgenic (TG) male littermates. The size of the testis is reduced in COX2 transgenic mice (n=4). (**B**) Cross-sections of testis from control (CT) and COX2 transgenic (TG) littermates at 25 weeks. While control testis contains sperms (arrows), sperms were not observed in COX2 transgenic testis. Scale bar=50 μm

**Figure 5:**
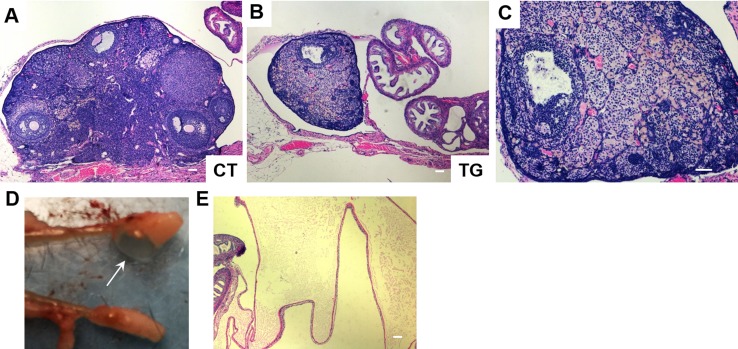
H&E staining of ovarian sections from 40-week-old (**A**) control (CT) and (**B**) COX2 transgenic mice (magnification x5). (**C**) Higher magnification (x10) view of the ovary shown in (**B**). (**D**) The ovary from 22-week-old COX2 transgenic mouse showing a cystic structure filled with a clear fluid (arrow). (**E**) H&E stained bursal cyst in COX2 transgenic mouse. The cyst contains pale eosinophilic proteinaceous material. Scale bar=100 μm.

Female fertility decreases with age, and males develop an age-associated decline in sperm count [36]. We set up matings between 10 COX2 transgenic males (12 ~ 23 weeks old) and 10 wild-type females. However, none of the wild type females became pregnant after two months of being housed together. Testes of the COX2 transgenic males were generally smaller than wild-type testes (Fig. 4A). Histological analysis of transgenic testes revealed the absence or reduced number of mature spermatozoa (Fig. 4B). We also set up matings between 12 COX2 transgenic females and 12 wild-type males. However, only one out of the 12 transgenic females became pregnant. Histological analysis showed that the ovaries of 40-week-old COX2 transgenic mice were atrophic with minimal follicles and compensatory interstitial hyperplasia, which suggests that these ovaries have been exhausted (Fig. 5A-C). Additionally, ovarian bursal cysts were often observed in COX2 transgenic female mice (Fig. 5D and E), which is commonly observed in aged mice [37].

**Figure 6:**
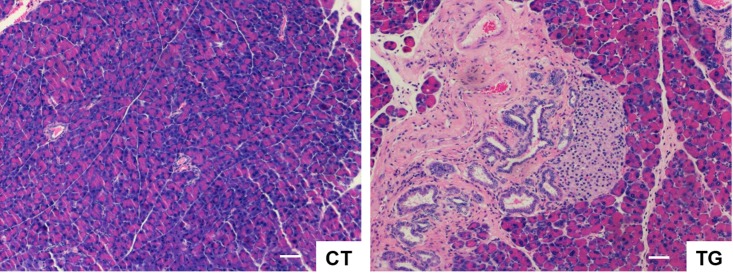
H&E stained sections of pancreas from COX2 transgenic mice (TG) showing the loss of the pancreatic acini, the increase of the metaplastic pancreatic ducts with dysplastic changes, peri-ductal fibrosis, and mononuclear inflammatory cell infiltration in the highly vascularized fibrotic stroma. Hyperplasia of the Langerhans islets was also noticed. Scale bar=100 μm.

The incidence and severity of pancreatitis increases in elderly population [38–40]. Colby et al. [41] have shown that bovine keratin 5 promoter-driven expression of COX2 results in dysplastic changes in the pancreas. Consistent with their findings, pancreata of COX2 transgenic mice exhibited phenotypes similar to chronic pancreatitis such as the loss of the pancreatic acini, the increase of the metaplastic pancreatic ducts with dysplastic changes, periductal fibrosis, and mono-nuclear inflammatory cell infiltration in the highly vascularized fibrotic stroma (Fig. 6). Hyperplasia of the Langerhans islets was also noticed, which is often associated with age in mice [42].

### Increased senescence in COX2 transgenic mice

**Figure 7:**
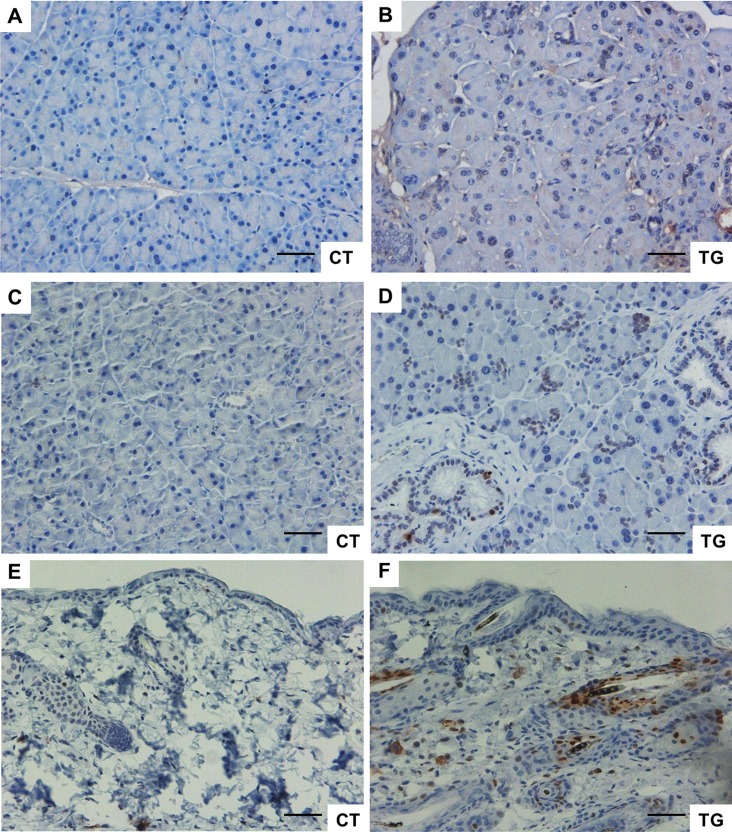
(**A**) and (**B**) Immunohistochemical staining of p16 in the pancreata from 25-week-old control and COX2 transgenic littermates (magnification x20). (**C**) and (**D**) Immunohistochemical staining of phospho-H2AX in the pancreata from 23-week-old control and COX2 transgenic littermates (magnification x20). (**E**) and (**F**) Immunohistochemical staining of phospho-H2AX in the skin from 25-week-old control and COX2 transgenic littermates (magnification x20). Scale bar=100 μm.

Cellular senescence has been proposed to contribute to aging phenotypes and/or the development of certain age-related diseases [43]. The number of senescent cells increases with age in mammalian tissues [44], and such cells have been found at sites of age-related pathologies such as osteoarthritis and atherosclerosis [45, 46]. Immunohistochemical analysis of p16*^Ink4a^*, an indicator of senescence, revealed that the number of p16*^Ink4a^*-positive cells was increased in the pancreata of COX2 transgenic mice compared to that in pancreata of control littermates (Fig. 7A and B). In addition, immuno-histochemical analysis of phospho-H2AX, a marker of DNA damage, showed that the number of phospho-H2AX-positive cells was increased in the pancreata of transgenic mice (Fig. 7C and D). Similarly, Western blot analysis showed increased levels of p16 and phospho-H2AX in transgenic pancreas (Supplementary Figure 1). Increase in the number of phospho-H2AX-positive cells was also observed in the skin of COX2 transgenic mice (Fig. 7E and F).

**Figure 8:**
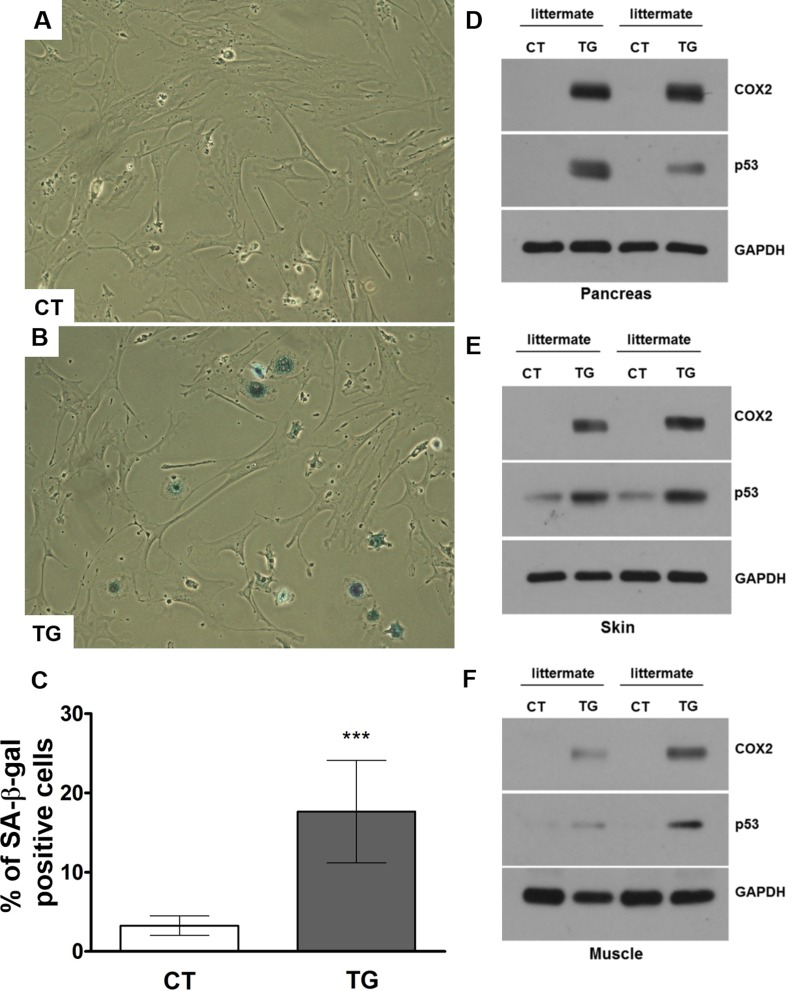
(**A**) and (**B**) Adult mouse lung fibroblasts from control and COX2 transgenic mice were incubated with 4-hydroxytamoxifen for 24 hours to induce COX2 expression in transgenic fibroblasts. The cells were cultured in the regular culture medium for 48 hours before SA-β-Gal staining. Representative pictures from 3 independent experiments are shown. Quantification results are shown in (**C**). (**D-F**) Western blot analysis of COX2 and p53 in pancreas (**D**), skin (**E**), and muscle (**F**) tissues of control and COX2 transgenic littermates.

To determine whether increased COX2 expression is associated with the increased cellular senescence, we performed senescence-associated beta-galactosidase (SA-β-gal) staining using adult mouse lung fibroblasts isolated from control and COX2 transgenic mice. The isolated fibroblasts were incubated with 1 μM of 4-hydroxytamoxifen to induce COX2 expression. As shown in Fig. 8A-C, the number of SA-β-gal-positive cells was increased in COX2 transgenic fibroblasts compared to that in control fibroblasts. However, treatment of COX2 transgenic fibroblasts with NS-398, a COX2 specific inhibitor, significantly reduced the number of SA-β-gal-positive cells (Supplementary Figure 2A and B). We previously have shown the up-regulation of p53 levels in COX2 transgenic embryos [24]. Moreover, we recently have demonstrated that COX2 regulates DNA damage-induced expression of p53 through modulation of oxidative stress [47]. To examine whether p53 expression is altered in COX2 transgenic mice, we assessed the levels of p53 in the tissues of control and transgenic mice. Western blot analysis revealed that expression of p53 was increased in the tissue lysates of COX2 transgenic mice (Fig. 8D-F). These results suggest that premature aging pheno-types may be linked to COX2-mediated induction of cellular senescence.

## DISCUSSION

Increasing evidence has implicated the role of COX2 in aging. Neonatal death and renal pathology in global COX2 knockout mice prevents them from being used to study the role of COX2 in the aging process [48]. We have developed a COX2 transgenic mouse model which allows conditional, tissue-specific COX2 expression [24]. Using this model, we have generated tamoxifen-inducible COX2 transgenic mice to investigate the biological significance of COX2 up-regulation during aging. When COX2 expression was induced in 5~6-week-old mice, COX2 transgenic mice developed various signs of aging such as reduced subcutaneous fat, delayed hair growth, chronic pancreatitis, infertility, and decreased muscle fiber size. In addition, the lifespan of COX2 transgenic mice was significantly reduced compared to that of wild type littermates. All COX2 transgenic mice exhibited at least some of these phenotypes characteristic of premature aging, implicating COX2 as a potential target in the control of age-related diseases.

Aging phenotypes observed in other models, such as liver pathologies and kyphosis were not observed in our model. This may be related to a low level of COX2 transgene expression in these tissues (data not shown). If this is the case, then it suggests that the effect of COX2 may be mediated by local production of prostanoids. Alternatively, COX2 may differentially affect the physiology of these tissues. For example, prostaglandin E_2_ (PGE_2_), a major prostanoid produced by COX2, is a strong inducer of bone formation in a variety of animals [49], including humans. In addition, COX2 has been shown to regulate mesenchymal cell differentiation into osteoblast lineage [50]. We have also recently shown that prostaglandin F_2ɑ_ (PGF_2ɑ_) stimulates chondrocyte differentiation and promotes bone morphogenetic protein (BMP) signaling [51]. Thus, increased expression of COX2 may protect the skeleton from age-associated damage such as osteo-porosis, which is frequently observed in other mouse models of aging. COX2 has been implicated in inflammatory and degenerative brain diseases [52]. In the current study, brain aging in COX2 transgenic animals was not analyzed. However, it has been shown that neuronal-specific COX2 transgenic mice develop age-dependent cognitive deficits and neuronal apoptosis [53], suggesting that COX2 also contributes to neuronal aging. Moreover, Mirjany et al. [54] have shown that COX2 accelerates glutamate-induced apoptotic damage in neuronal-specific COX2 transgenic mice.

It is well-known that COX2 plays an important role in inflammation. Chronic inflammation is a hallmark of aging and promotes many age-related diseases [55, 56]. It has been shown that knockout of the *nfkb1* subunit of the transcription factor NF-κB causes chronic inflammation and accelerated aging [57]. In the same study, ibuprofen, a general COX inhibitor, reduced inflammation and restored regenerative capacity of hepatocytes in *nfkb1*^−/−^ mice. However, although COX2 expression caused pancreatitis in our COX2 transgenic model, the signs of chronic inflammation such as increased tissue infiltration of immune cells and elevated levels of inflammatory cytokines were not observed. This suggests that chronic inflammation may not be the mechanism by which COX2 regulates the aging process. Currently, how COX2 induces aging phenotypes is not clear. At the cellular level, COX2 inhibitors suppressed the replicative senescence, while PGE_2_ promoted cellular senescence [58–60], suggesting the involvement of prostaglandins and their receptors in cellular senescence. However, another study has shown that celecoxib, a COX2 specific inhibitor, extends the life span of *C. elegans* and delays the age-associated physiological changes via inhibition of insulin-like signaling, but not via COX2 activity [61]. On the other hand, a mouse study has shown that generation of reactive oxygen species (ROS) increases with age, which may result from increased COX2 expression and activity in aged animals [62].

p53 is known to play a pivotal role in cellular homeostasis; thus, dysregulation of p53 signaling is linked to aging or to the development of diseases such as cancer. Expression of p53 is induced by various cellular or environmental stimuli. Intriguingly, many signals that activate p53 are known to induce COX2 expression as well [63], suggesting the existence of cross-talk between these two pathways. It is well-known that p53, as a transcription factor, positively or negatively regulates COX2 expression. However, the role of COX2 as an upstream regulator of p53 has not been well-studied. We previously have demonstrated that COX2 positively regulates p53 levels [24]. In COX2 transgenic embryos which develop severe axial skeletal malformations, accumulation of p53 protein was dramatically increased in the precursor cells of the axial skeleton, indicating that COX2 functions as an upstream regulator of p53 signaling. Moreover, we recently have shown that doxorubicin-induced p53 expression is reduced by inhibition or knockdown of COX2, further supporting the role of COX2 in regulating p53 [47]. Although the underlying mechanism by which COX2 causes elevated levels of p53 warrants further study, previous reports suggested that COX2 can regulate p53 through prostaglandin-dependent and –independent mechanisms. For example, it has been shown that PGE_2_ stimulates p53 activity in human synovial fibroblasts through p38 kinase-mediated phosphorylation of p53 [64]. Additionally, PGE_2_ has been shown to be involved in p53 activation and maintenance of the senescent phenotype in chronic obstructive pulmonary disease (COPD) fibroblasts [65]. On the other hand, COX2 has been shown to induce genomic instability [66] and generate reactive oxygen species [67] in a prostaglandin-independent manner. In the current study, p53 expression was up-regulated in the tissues of COX2 transgenic mice, suggesting that COX2-mediated p53 activation may contribute to premature aging phenotype. Future study with p53 null mice will determine whether aging-phenotypes in COX2 transgenic mice are p53-dependent.

COX2 expression is increased in many age-related human diseases and in the tissues of aged humans and mice, implicating the involvement of COX2 in the aging process. However, the biological significance of increased COX2 expression during aging has not been determined. Our data suggest that targeting of COX2 and its downstream pathways may have therapeutic and preventive potential against aging and age-related diseases.

## MATERIALS AND METHODS

### Generation of COX2 transgenic mice

All animal studies and procedures were approved by the University of South Carolina Institutional Animal Care and Use Committee. The transgenic basic cassette, pCAG-CAT-HES-poly(A), was a gift from Dr. Junichi Miyazaki (Osaka University Medical School, Japan). Human COX2 cDNA was inserted into HindIII and EcoRV sites of pCAG-CAT-HES-poly(A). The transgenic vector was digested with SalI and PstI to remove the vector region. The insert fragment was recovered from the gel and diluted to 2 μg/ml concentration in 1 mM Tris/HCl (pH 8.0) and 0.1 mM EDTA. The DNA fragment was introduced into pronuclei of 0.5-day-old mouse embryos (B6D2F1, Taconic) by glass capillaries. Injected embryos were cultured in KSOM (Sigma) for 1 day, and embryos that reached the two-cell stage were transferred into oviducts of pseudopregnant females. The offspring were initially screened by PCR for the chloramphenicol acetyltransferase (CAT) gene from tail tissue (CAT2 primer, 5′-CAGTCAGTTGCTCAATGTACC-3′; CAT3 primer, 5′-ACTGGTGAAACTCACCCA-3′). For production of the CAT^fl^COX2 mice, five lines were initially established, and two of them, lines 12 and 17, showing high CAT activity in the liver, were chosen for further analysis. ROSA-Cre ERT2 mice were obtained from the Jackson Laboratory. A ROSA-Cre ERT2 female (or CAT^fl^COX2 female) mouse was housed overnight with a CAT^fl^COX2 male (or ROSA-Cre ERT2 male) mouse.

### Genotyping and tamoxifen treatment

Genomic DNA was isolated from tails using a DNeasy kit (Qiagen). Isolated genomic DNA was amplified using ExTaq DNA polymerase (Takara) with a primer set designed to detect the presence of a recombined human COX2 allele (forward primer, 5′-GTGCTGGTTATTGTGCTGTCTC-3′; reverse primer, 5′-TCTCCATAGAATCCTGTCCGGGTA-3′), and PCR products were run on 1.2 % agarose gel. The recombined human COX2 allele was identified as a ∼300 bp PCR product, whereas the non-recombined human COX2 allele was identified as a ∼1.8 kb fragment. To detect the presence of the Cre gene, genomic DNA was amplified by PCR using a primer set for Cre (forward primer, 5′-ACCTGAAGATGTTCGCGATTATCT-3′; reverse primer, 5′-ACCGTCAGTACGTGAGATATCTT-3′). In order to induce COX2 expression, 100 μl of tamoxifen (10 mg/ml in corn oil) was intra-peritoneally injected every 3 days, for a total of three times, to 5-week-old control (wild type, CAT^fl^COX2, or Rosa-CreERT2) and transgenic (CAT^fl^COX2:Rosa-CreERT2) mice.

### Histology and immunohistochemistry

Mouse tissues were collected, fixed in 10% neutral buffered formalin, and embedded in paraffin blocks. Tissue sections (5 μm) were subjected to hematoxylin-eosin (H&E) staining by standard procedures. For immunohistochemical analysis, antigen retrieval was performed by immersing sections in 10 mM sodium citrate (pH 6.0) at 95°C for 30 min. Sections were incubated with primary antibody [human COX2 (1:500), Cayman Chemical; p16 (1:500), Santa Cruz Biotechnology; cleaved caspase-3 (1:250), Cell Signaling] at 4°C overnight. For detection of phospho-Histone H2AX, sections were subjected to EDTA (1 mM, pH 8.0) antigen retrieval for 20 min and incubated with primary antibody [phospho-Histone H2AX (Ser139) (1:500), Cell Signaling] at 4°C overnight. Washed sections were incubated in the ImmPress reagent (Vector Laboratories) for 30 min and visualized with diaminobenzidine. After mounting, the sections were observed under an Oympus BX51 light microscope, and the image was acquired by an AxioCam MRc camera.

### Isolation of mouse lung fibroblasts and SA-β-galactose staining

Isolation of adult mouse lung fibroblasts from control and transgenic mice was carried out as described [68]. For SA-β-galactose staining, 5 × 10^5^ fibroblasts were plated in 60 mm culture dishes, and incubated with 1 μM 4-hydroxytamoxifen (Sigma) for 24 hours. Forty-eight hours after 4-hydroxytamoxifen treatment, the cells were fixed in 2 % formaldehyde/0.2 % glutaraldehyde, washed with phosphate-buffered saline, and incubated at 37°C overnight in stain solution [1 mg/ml 5-bromo-4-chloro-3-indolyl-β-D-galactopyrano-side (X-gal), 40 mM citric acid-sodium phosphate (pH 6.0), 150 mM NaCl, 2 mM MgCl_2_, 5 mM potassium ferrocyanide, 5 mM potassium ferricyanide].

### Western blot analysis

Tissues were lysed in radioimmune precipitation buffer, and protein concentration was measured using a BCA protein assay kit (Pierce). Equal amounts of protein were heated at 65°C in LDS sample buffer (Invitrogen) with sample reducing agent (Invitrogen) for 10 min and then separated by SDS-PAGE. The separated proteins were transferred to an Immobilon-P membrane (Millipore). Following incubation in blocking buffer (TBS with 5% nonfat dry milk and 0.1% Tween 20) for 1 hour at room temperature, the membranes were incubated with primary antibodies [p53 (1:2000), Cell Signaling; GAPDH (1:5000), Cell Signaling; human COX2 (1:500), Cayman Chemical] diluted in blocking buffer overnight at 4°C. The membranes were washed and then probed with a horseradish peroxidase-linked secondary antibody (Cell Signaling) for 1 hour at room temperature. Detection was made with an enhanced chemiluminescence reagent (GE Healthcare Life Sciences), followed by exposure of membrane to film.

### Statistics

Statistical analysis was performed using Prism software (Graphpad Software, La Jolla, CA). Values of p≤ 0.05 were considered statistically significant.

## SUPPLEMENTARY MATERIAL FIGURES


