# Supplementary material for: Transgenic expression of cyclooxygenase-2 (COX2) causes premature aging phenotypes in mice
Source: Aging (Albany NY). 2016 Oct 7;8(10):2392–405. doi: 10.18632/aging.101060 (PMC5115895; doi:10.18632/aging.101060)
Supplement: Supplementary file 1 [file aging-08-2392-s001.pdf]

SUPPLEMENTARY MATERIAL

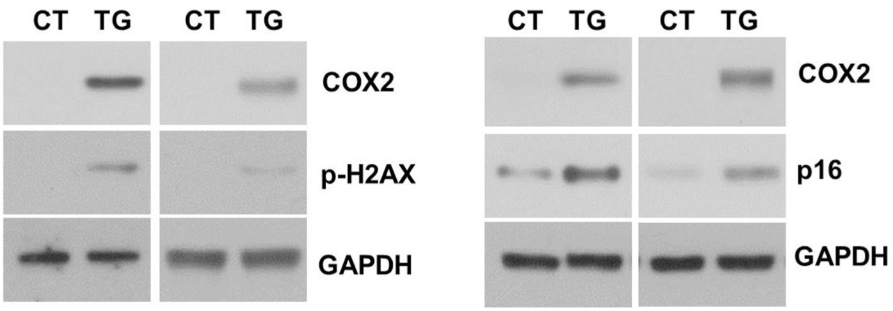

Supplementary Figure 1. Western blot analysis of COX2, phospho-H2AX, and p16 in pancreas of control (CT) and COX2 transgenic (TG) mice.

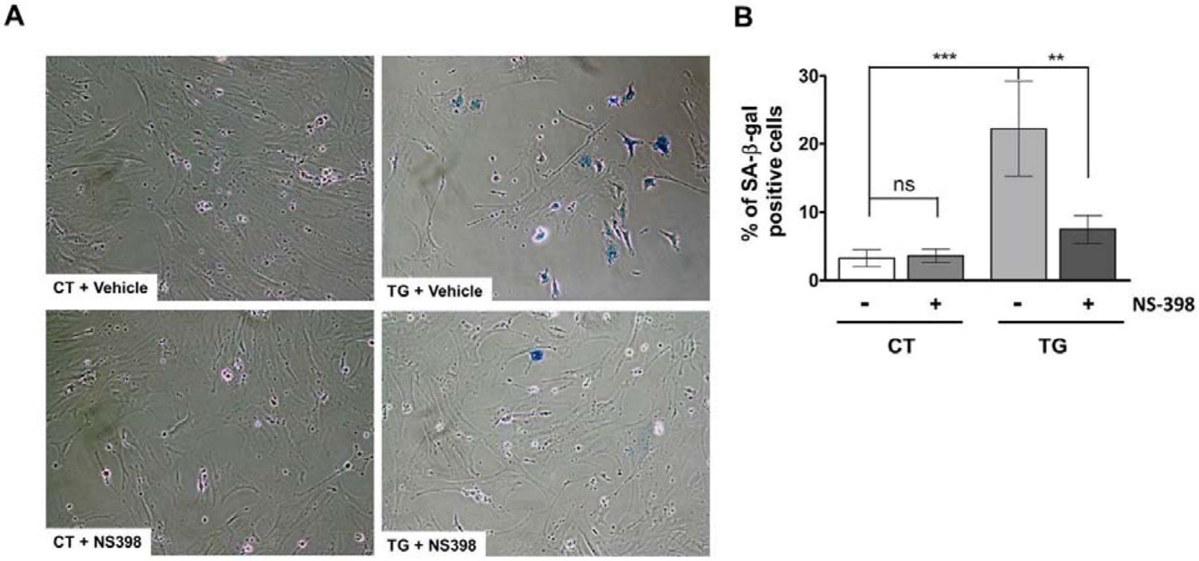

Supplementary Figure 2. (**A**) Adult mouse lung fibroblasts from control and COX2 transgenic mice were incubated with 4-hydroxytamoxifen for 24 hours to induce COX2 expression in transgenic fibroblasts. The cells were cultured in the regular culture media containing DMSO or 10 μM NS398 for 72 hours before SA-β-Gal staining. Quantification results are shown in (**B**).
